# Epigallocatechin Gallate Attenuates Bladder Dysfunction via Suppression of Oxidative Stress in a Rat Model of Partial Bladder Outlet Obstruction

**DOI:** 10.1155/2018/1393641

**Published:** 2018-07-22

**Authors:** Meng Gu, Chong Liu, Xiang Wan, Tianye Yang, Yanbo Chen, Juan Zhou, Qi Chen, Zhong Wang

**Affiliations:** ^1^Department of Urology, Shanghai Ninth People's Hospital Affiliated to Shanghai Jiao Tong University School of Medicine, Shanghai 200011, China; ^2^Department of Emergency, Shanghai Ninth People's Hospital Affiliated to Shanghai Jiao Tong University School of Medicine, Shanghai 200011, China

## Abstract

**Purpose:**

To investigate the protective effect of epigallocatechin gallate (EGCG), a green tea extract, and its underlying mechanism on bladder dysfunction in a rat model of bladder outlet obstruction (BOO).

**Materials and Methods:**

Sprague-Dawley rats of BOO were surgically induced and followed by treatment with EGCG (5 mg/kg/day) or saline (control) via intraperitoneal injection. Cystometry was performed on four weeks postoperatively in conscious rats. H&E, Masson trichrome, and TUNEL staining were performed to observe tissue alterations. Oxidative stress markers were measured, and protein expression of Nrf2-ARE pathway was examined by immunohistochemistry and Western blotting.

**Results:**

Our data showed that EGCG could increase the peak voiding pressure and bladder compliance and prolong micturition interval of BOO rats compared with control and finally reduce the frequency of urinary. EGCG could ameliorate the increase of collagen fibers and ROS induced by obstruction and increase the activity of SOD, GSH-Px, and CAT. The level of cell apoptosis was decreased in BOO rats treated with EGCG compared with control, and caspase-3 expression was reduced as well. Moreover, EGCG could activate the Nrf2 expression with elevation of its target antioxidant proteins.

**Conclusions:**

EGCG alleviates BOO-induced bladder dysfunction via suppression of oxidative stress and activation of the protein expression of Nrf2-ARE pathway.

## 1. Introduction

Benign prostatic hyperplasia (BPH) characterized by gradually nonmalignant enlargement of prostate gland [1, 2] is a very common chronic disease in elderly men. As people age, most BPH patient will induce bladder outlet obstruction (BOO) with high bladder pressure and low flow [3]. BOO is a common disorder of the urinary tract that could affect quality of life and is a very rarely life-threatening disease but can induce significant structural and functional changes of the bladder, which in turn brings lower urinary tract symptoms (LUTS) including urinary frequency, urgency, nocturia, and urge incontinence [4]. BOO caused by BPH has become increasingly prevalent with age and been considered as the major public health problem that seriously affects the quality of life of patients and their partners.

As showed in previous studies, oxidative stress is considered to be one of the mechanisms that triggers reactions chain involved in the development and progression of BPH and leads to injured function of the bladder [5]. Oxidative stress occurs in the cellular environment when there is an imbalance between the production of reactive oxygen species (ROS) and the ability of biological systems to repair oxidative damage or neutralize the effects of reactive intermediates including peroxides and free radicals. Production of high levels of ROS causes a significant decrease in antioxidant defense mechanisms leading to protein, lipid, and DNA damage and subsequent disruption of cellular functions and cell death but at lower levels induce subtle changes in intracellular signaling pathways. An increase in ROS may contribute to alter bladder function in aging [6]. Superoxide dismutase (SOD) is one of the cell's chief defenses against activated oxygen free radicals. Presenting in the peroxisomes of nearly all aerobic cells, catalase (CAT) act in association with SOD protects cells against free radical damage [7, 8]. SOD and CAT activities are associated with the shift from compensated to decompensated function of the bladder [7]. As a transcription factor, nuclear erythroid-related factor 2 (Nrf2) could promote expression of antioxidative genes through the antioxidant response element (ARE) to regulate cellular antioxidative responses and redox status [9]. It has been established in the literature that activation of the Nrf2-ARE pathway may ameliorate bladder dysfunction caused by bladder outlet obstruction [10].

As a major component of green tea, epigallocatechin-3-gallate (EGCG) has been studied for its antioxidative and anti-inflammatory properties. Reports revealed that pretreatment with EGCG could induce Nrf2 activation in cells as demonstrated by increasing expression and nuclear translocation of Nrf2 [11, 12]. Mechanical stretch and hypoxia have been reported to contribute to the functional and structural changes in the bladder after BOO, and previous studies have indicated that the activation of Nrf2 pathway by EGCG shows effective protection in various diseases. However, whether EGCG could protect bladder tissue by activating the Nrf2 pathway in the BOO model has not been well defined. Based on our previous study [10] that demonstrated the decrease of oxidative stress and activation of the Nrf2-ARE pathway may ameliorate bladder dysfunction induced by BOO, we explored the ability of EGCG to ameliorate bladder dysfunction by inhibiting oxidative stress via the regulation of the Nrf2-ARE pathway in a rat model of BOO in the present study.

## 2. Materials and Methods

### 2.1. BOO Model and Drug Treatment

BOO model of SD was established in adult male Sprague-Dawley rats (SD) weighing 240 to 260 g (Animal Center of the Shanghai Ninth People's Hospital of Shanghai Jiao Tong University School of Medicine, Shanghai, China) according to our previous method [10]. All animals were housed by two per cage in a room under controlled temperature, humidity, and 12-hour light/12-hour dark cycles with free access to food and water. The rats were allowed to acclimatize for at least five days before the experiment. All experimental procedures were approved by the Ethics Committee of Shanghai Jiao Tong University School of Medicine. The SD rats were randomly divided into three groups: the sham-operated group, the BOO group, and the BOO treated with EGCG (purity > 98%, Biotech Co. Ltd, China) group, and 8 rats were included in each group. BOO surgical operation was applied according to the method described previously in detail [13]. Briefly, the rats were anesthetized and then abdominal midline incision (about 1.0 cm) was made to expose the bladder and proximal urethra. The proximal urethra was loosely tied with the needle using 3–0 silk thread after a 19-G needle was placed around it after which the needle was removed and incision was closed. Sham operations were performed in an identical manner without tying the silk thread.

From the first day after operation, rats of the BOO + EGCG group received daily intraperitoneally injections of EGCG (5 mg/kg). EGCG was dissolved in PBS, and rats of the sham group and BOO group were given the same volume of PBS.

### 2.2. Cystometry Preparation and Cystometric Analysis

The cystometry preparation and cystometric analysis were performed four weeks after operation. A catheter was placed in the bladder of rats of all groups three days before cystometric analysis. As described in previous study, flaring the end of the polyethylene tubing 50 (PE-50) to become a balloon serves as an anchor to maintain the tube within the bladder [7, 11]. A 1 cm incision was made on the dorsum between the scapulas in anesthetized rats followed by developing a plane between the skin and the underlying muscle to create a tunnel around the ventral abdomen. Cystometry preparation was carried out as followed that rats were anesthetized and a 1 cm incision was made on the dorsum between the scapulas. Then, we developed a plane between the skin and the underlying muscle to create a tunnel around the ventral abdomen. After an abdominal midline incision was made to expose the bladder, we grasped the smooth end of the PE-50 tubing with the clamp and pull it back through the dorsum incision. Following the bulbed end was placed in the bladder dome and the purse string suture around the tubing was pulled tight, the dorsal and abdominal incisions were closed for cystometric analysis.

For cystometry, the conscious rats were placed in a metabolic cage, and the indwelling tubing was attached to a two-way valve that was connected to a pressure transducer as well as an infusion pump. Saline was infused into the bladder at a rate of 12 ml/h at room temperature in all groups. The cystometric parameters of maximal pressure, bladder capacity, and others were measured. The rats were euthanized after the experiment, and the bladder tissue was collected for further study.

### 2.3. Histological Examination and Immunohistochemical Staining

5 *μ*m sections were made after bladders being fixed in 4% paraformaldehyde and embedded in paraffin. Hematoxylin and eosin (H&E) staining was performed to observe general morphology of the bladders, and Masson trichrome staining was used to evaluate the level of tissue fibrosis. Immunohistochemical staining was also conducted as previously described in our study. Briefly, the sections were subjected with 10 mM sodium citrate buffer (pH 6.0) to heat for antigen retrieval. The primary antibodies of anti-Nrf2 (Abcam, Cambridge, UK), HO-1 (Abcam, Cambridge, UK), PCNA (CST, Danvers, MA, USA), secondary antibodies of goat-anti-rabbit IgG-HRP (DAKO, Denmark), goat-anti-mouse IgG-HRP (DAKO, Denmark), and DAB detection kit (DAKO, Denmark) were used according to the manufacturer's instructions. Histological analysis was performed by a pathologist in a blinded manner.

### 2.4. Apoptosis Assay by TUNEL

The apoptosis level in the bladder smooth muscle was measured by the one-step TUNEL apoptosis assay kit (KeyGen Biotech, Nanjing, China) according to the manufacturer's instructions. In brief, the sections were regularly hydrated and immersed in 1% Triton X-100 followed by being incubated with proteinase K solution for 30 minutes. And the sections were reacted with TdT solution for 1 hour and then streptavidin-TRITC solution for 30 minutes in a humidified and dark chamber, respectively. Finally, DAB detection kit was used to stain the sections to observe and analyze apoptotic cells by a pathologist in a blinded manner.

### 2.5. MDA, GSH-Px, Total SOD (tSOD), ROS, and CAT Determination

According to our previous study [10], we choose some marker routine laboratory indexes of oxidative stress as malondialdehyde (MDA), glutathione peroxidase (GSH-Px), total superoxide dismutase (tSOD), and catalase activity (CAT) were conducted. The content of MDA, GSH-Px, total tSOD, and CAT in the bladder tissues was measured according to the manufacturer's protocols of the assay kit of MDA, tSOD, GSH-Px, and CAT (Nanjing Jiancheng Bioengineering Institute, Nanjing, China). Briefly, the maximum absorbance was at 532 nm based on thiobarbituric acid (TAB) method to detect MDA level, the tSOD activity was measured based on the combination of xanthine and xanthine oxidase and the absorbance was read at 550 nm, and the GSH-Px activity assay was performed using the enzyme-catalyzed reaction product (reduced glutathione) and the absorbance was recorded at 412 nm. To measure the CAT activity, hydrogen peroxide reacted with ammonium molybdate, and the absorbance at 405 nm was then detected. Considering accumulation of reactive oxygen species (ROS) occurs and leads to functional alterations and pathological conditions such as a bladder dysfunction developed with age [6, 14, 15]; ROS was also detected in our study. According to the procedure of the ROS assay kit (Sigma-Aldrich, MO, USA), DCFDA was used as fluorescent probe following by washing using assay buffer. Immediately after the excitation at a wavelength of 485 nm, the absorbance is read at 535 nm.

### 2.6. Western Blotting

Total protein was extracted from frozen bladder tissues selected randomly from each group (*n* = 3) by trypan blue in RIPA buffer containing protease inhibitors. Muscle lysates were centrifuged at 10000 ×g/min at 4°C for 10 min, and the supernatant was collected and used as total protein extracts. The nuclear protein was extracted according to the manufacturer's instructions of nuclear protein extraction kit (Beyotime Institute of Biotechnology, Shanghai, China). Following the steps outlined below, bladder tissues were cut in pieces and homogenized in cytoplasm protein extraction reagent containing protease inhibitors, the lysates were centrifuged (10000 ×g/min) at 4°C for 5 min, and then the precipitation was collected and mixed with nucleoprotein extraction reagent for 30 min on ice; after the lysates were centrifuged (10000 ×g/min) at 4°C for 10 min, the supernatant was collected and used as nuclear protein extracts. Protein concentrations were measured using BCA protein assay kit (Thermo Scientific, MA, USA). The samples were run on 10% SDS-polyacrylamide gels (20 *μ*g/lane), and then, proteins were transferred to PVDF membranes by electroblotting (200 mA). PVDF membranes were incubated in 5% BSA for 2 hours at room temperature, followed by three 5 minutes washing in TBST. The PVDF membranes were then incubated overnight at 4°C with anti-Nrf2 (1 : 1000), HO-1 (1 : 1000), NQO1 (1 : 1000), Caspase-3 (CST, Danvers, MA, USA) (1 : 1000), GAPDH (CST, Danvers, MA, USA) (1 : 2000), and Lamin-B (CST, Danvers, MA, USA) (1 : 2000) antibodies. GAPDH and Lamin-B were used as internal normalizer. Then, the membranes were washed for 10 min three times in TBST and incubated with IgG-HRP antibody (1 : 2000) for 2 hours and finally examined by chemiluminescence.

### 2.7. Statistical Analysis

The values were presented as mean ± SD. SPSS 17.0 was used to evaluate the difference. Differences between groups were analyzed using one-way ANOVA with *P* < 0.05 considered significant.

## 3. Results

### 3.1. EGCG Ameliorated Voiding Dysfunction Induced by BOO

**Figure 1:**
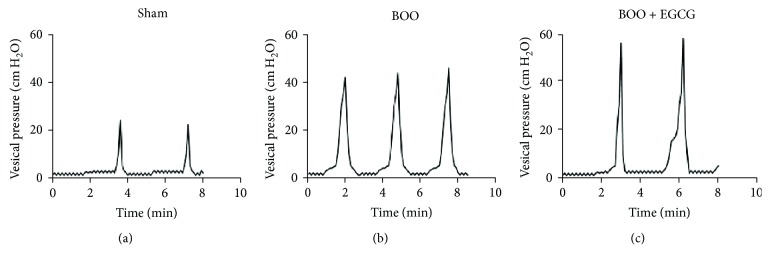
Effect of EGCG on urodynamic changes in conscious rats.

The cystometry results in each group are showed in Table 1 which were obtained according to the urodynamic curve (Figure 1). The voiding pressure was significantly increased in obstructed rats compared with sham rats. The peak voiding pressure in obstructed rats with EGCG treatment is higher than obstructed rats at the 4-week time point. Bladder capacity was significantly higher in BOO rats compared with sham rats. Rats in the BOO + EGCG group showed the highest bladder capacity among all groups. Bladder compliance decreased significantly at the 4-week time point after BOO. EGCG may rescue the compliance deterioration through increasing bladder capacity. It was found that the interval of micturition was shorter in BOO rats than in sham rats, and the micturition interval in BOO + EGCG rats was significantly increased compared to BOO rats.

### 3.2. Effect of EGCG in Bladder Detrusor of BOO Rats on Histological Changes

**Figure 2:**
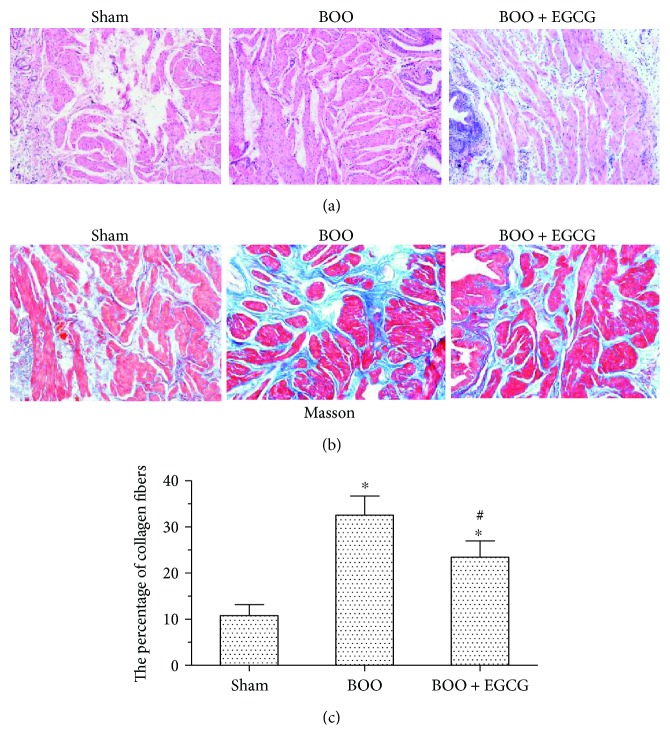
Effect of EGCG on bladder histological changes in BOO rats. Original magnification ×100. H&E (a) and Masson trichrome (b) staining in the sham, BOO, and BOO + EGCG bladders and (c) the percentage of collagen fibers in muscular layer in the sham, BOO, and BOO + EGCG bladders; ^∗^*P* < 0.05, *n* = 6 versus the sham group; ^#^*P* < 0.05, *n* = 6 versus the BOO group.

As showed in Figure 2(a), EGCG had some protective effect on BOO bladder based on H&E staining. BOO caused obvious histological changes, such as the structural damage of detrusor smooth muscle while treatment with EGCG significantly alleviated these histological changes in the bladders of BOO rats. The area ratio of collagen fibers was 10.77 ± 2.39, 32.53 ± 4.15, and 23.41 ± 3.53 in the sham group, BOO group, and BOO + EGCG group, respectively (Figure 2(c)). EGCG suppressed collagen fibers in the BOO + EGCG group as compared with that in the BOO group (Figure 2(b)).

### 3.3. EGCG Attenuated Oxidative Stress in the Bladder of BOO Rats

**Figure 3:**
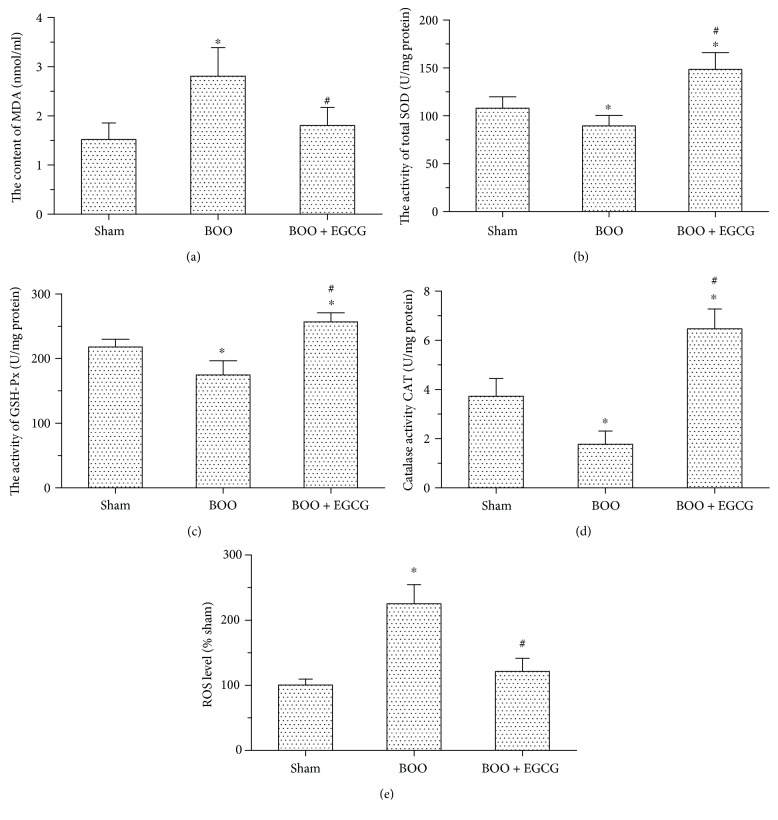
Effect of EGCG on oxidative stress in the bladder of BOO rats. (a) MDA level in the bladder of the three groups, ^∗^*P* < 0.05 versus the sham group, *n* = 6; ^#^*P* < 0.05 versus the BOO group, *n* = 6. (b) The activity of total SOD in the bladder of the three groups, ^∗^*P* < 0.05 versus the sham group, *n* = 6; ^#^*P* < 0.05 versus the BOO group, *n* = 6. (c) The activity of GSH-Px in the bladder of the three groups, ^∗^*P* < 0.05 versus the sham group, *n* = 6; ^#^*P* < 0.05 versus the BOO group, *n* = 6. (d) The activity of CAT in the bladder of the three groups, ^∗^*P* < 0.05 versus the sham group, *n* = 6; ^#^*P* < 0.05 versus the BOO group, *n* = 6. (e) The relative activity of ROS in the bladder of the three groups, ^∗^*P* < 0.05 versus the sham group, *n* = 6; ^#^*P* < 0.05 versus the BOO group, *n* = 6.

To evaluate the level of oxidative stress, we measured the content of MDA, SOD, GSH-Px, CAT activity, and ROS. As showed in Figure 3(a), the content of MDA in the bladder was significantly increased in BOO rats compared with sham rats (2.81 ± 0.58 versus 1.52 ± 0.33). Compared with BOO rats, EGCG treatments significantly inhibited MDA level increase induced by BOO (1.81 ± 0.37 versus 2.81 ± 0.58). The activities of tSOD and GSH-Px had some content of decrease in BOO rats compared with sham rats (89.65 ± 10.70 versus 108.06 ± 11.88; 174.71 ± 11.68 versus 218.18 ± 12.44), respectively. However, EGCG treatment could significantly increase the tSOD and GSH-Px activities in BOO rats (Figures 3(b) and 3(c)). As compared with sham rats, CAT activity significantly decreased in the BOO group (*P* < 0.05), which was upregulated by EGCG, even compared with the sham group (Figure 3(d)). In contrast to CAT, ROS was increased in the BOO group as compared with the sham group, and EGCG decreased it significantly (*P* < 0.05) (Figure 3(e)).

### 3.4. EGCG Inhibited Cell Apoptosis and Prompted Proliferation in the Bladder of BOO Rats

**Figure 4:**
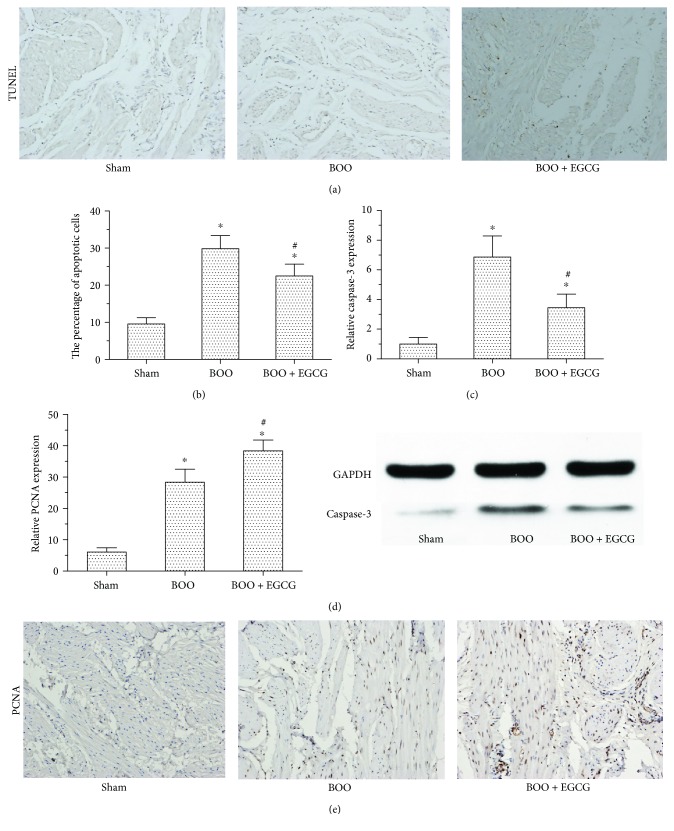
Effect of EGCG on cell apoptosis and proliferation in BOO rats. (a) TUNEL staining showed the cell apoptosis level of the bladder in all the three groups. (b) The statistical results of TUNEL staining in the three groups. ^∗^*P* < 0.01, *n* = 6 versus the sham group; ^#^*P* < 0.01, *n* = 6 versus the BOO group. (c) The protein expression of caspase-3 in the bladder of the three groups. (d-e) The statistical results of PCNA in the bladder of the three groups using immunohistochemical staining to show cell proliferation. ^∗^*P* < 0.05 versus the sham group; ^#^*P* < 0.05 versus the BOO group. Original magnification ×200 for TUNEL and immunohistochemical staining.

TUNEL staining was performed to explore the effect of EGCG on the cell apoptosis of the bladder in BOO rats. The number of apoptotic cells in the bladder of BOO rats was markedly increased compared with sham rats (*p* < 0.01), whereas EGCG treatment significantly decreased the number of apoptotic cells in the bladder of BOO rats (*p* < 0.05) (Figures 4(a) and 4(b)). Western blotting showed that the caspase-3 expression was significantly increased in the bladder of BOO rats; however, the expression of caspase-3 was decreased in BOO + EGCG rats (Figure 4(c)) which was in accordance with the result of TUNEL staining. Moreover, the expression of PCNA measured by the immunohistochemical staining showed that EGCG caused significantly increase on cell proliferation in the BOO + EGCG group compared with the BOO group (Figure 4(d)).

### 3.5. EGCG Affected Protein Expression of the Nrf2-ARE Pathway

**Figure 5:**
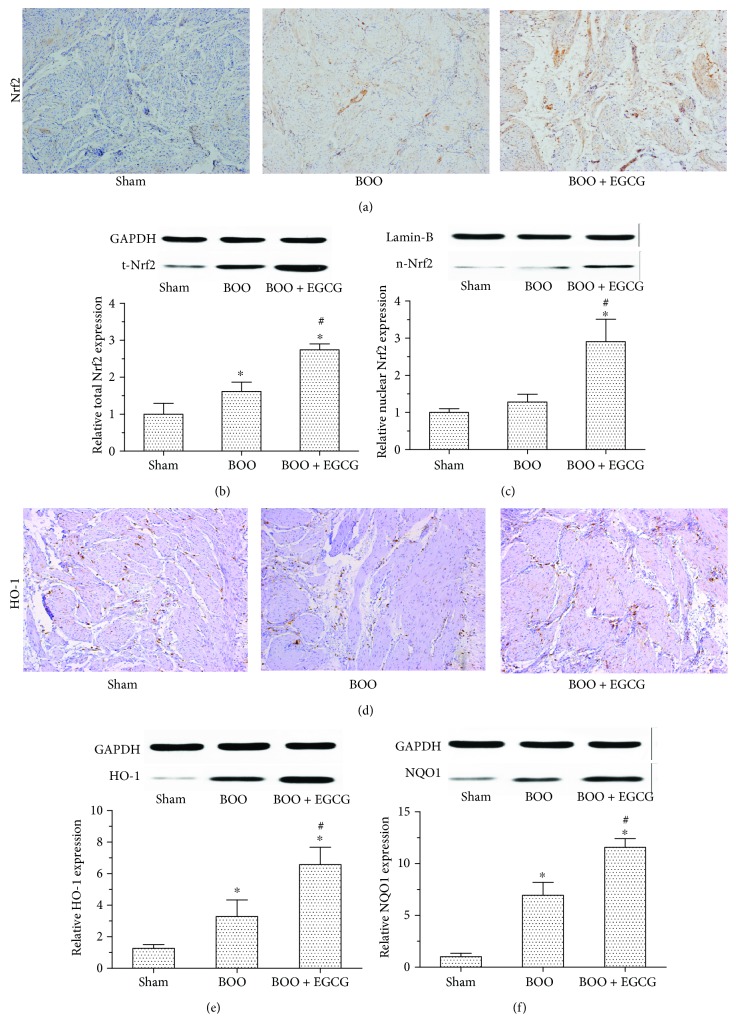
Effect of EGCG on protein expression of the Nrf2-ARE pathway. (a) The immunohistochemical staining of Nrf2. Protein expression of total Nrf2 (t-Nrf2) and nuclear Nrf2 (n-Nrf2) in the bladder of the three groups measured by Western blotting is shown in (b) and (c). The protein expression of HO-1 measured by immunohistochemical staining and Western blotting in the bladder of the three groups is shown in (d) and (e). (e) The protein expression of NQO1 in the bladder of the three groups. ^∗^*P* < 0.05 versus the sham group; ^#^*P* < 0.05 versus the BOO group.

Considering that Nrf2 is a vital mediator in regulating cellular antioxidative response, we explored the effect of EGCG on Nrf2 and its downstream target proteins in our study. The expression of Nrf2 measured by immunohistochemical staining was significantly higher in the muscular layers of the bladder in the BOO + EGCG group compared with the BOO group (Figure 5(a)). The expression of Nrf2 in the cell (t-Nrf2) and nucleus (n-Nrf2) was also measured by Western blotting. It is shown in Figures 5(b) and 5(c) that Nrf2 expression (t-Nrf2 and n-Nrf2) was significantly increased in the bladder of the BOO + EGCG group compared with the BOO group, and the t-Nrf2 was increased in the bladder of the BOO group compared with the sham group. It was worth noting that our results demonstrated the expression of Nrf2 was mainly located in the nucleus of the bladder cells while the expression of Nrf2 in the nucleus showed no significant difference between the BOO group and the sham group. We then detected the expression of Nrf2's downstream target proteins as HO-1 and NOQ1 to investigate its antioxidative function. Our data indicated that the expression of HO-1 was increased in the BOO + EGCG group compared with the BOO group, which was consistent with the result of n-Nrf2 expression (Figures 5(d) and 5(e)). The level of NQO1 in the BOO group was higher than that in the sham group, but lower than that in the BOO + EGCG group (Figure 5(f)).

## 4. Discussion

As investigated in animal models, chronic bladder ischemia might produce oxidative leading to denervation of the bladder and the expression of tissue-damaging molecules in the bladder wall, which could be responsible for the development of bladder hyperactivity progressing to bladder underactivity [16, 17]. It is also suggested that oxidative stress plays a critical role in bladder outlet obstruction-mediated bladder dysfunction [16, 17]. BOO-induced bladder remodeling in a rat model is similar to that in patients with BPH who suffered from bladder outlet obstruction. It was showed in our previous study that rats of BOO model were useful models to explore structural and functional alterations in the bladder [10]. Therefore, exploring good ways to reduce oxidative stress of bladder caused by BOO has been considerably attractive.

EGCG is a major catechin in green tea with functions of antioxidant, antiproliferative, anti-inflammatory, and attenuating metabolic syndrome, without adverse effects [18, 19]. Hsieh et al. reported that EGCG can attenuate the prostate enlargement in BPH rats accompanied with metabolic syndrome induced by high-fat diet combined with testosterone injection [10]. For human, according to reports in the literature, ten days' repeated administration of oral doses of EGCG of up to 800 mg per day was found to be safe and very well tolerated. The content of EGCG in green tea is about 10%. No obvious side effects occur in the amount of 20 cups of green tea per day [20]. In the present study, we investigated the effect of EGCG on BOO-induced bladder dysfunction in vivo and the daily intraperitoneal injection dose (5 mg/kg EGCG) was used which was similar to the dose that has been proved effective in other's study [10, 21].

We performed urodynamic studies in conscious rats of BOO to accurately reflect the real situation. The cystometric parameters such as bladder capacity, maximal pressure, compliance, and micturition interval were measured; we found that the micturition interval, capacity, and compliance were significantly increased, and the peak voiding pressure had some extent of increase by treatment with EGCG in BOO rats. The histological staining showed that bladder smooth muscle bundles were severely damaged and collagen deposition was increased in BOO rats. This increase of collagen deposition was suppressed by EGCG treatment for 4 weeks in BOO model rats. Our results indicated that there is an inverse relationship between bladder compliance and collagen fibers, and EGCG could protect bladder from BOO-induced dysfunction and morphological damage.

In order to investigate antioxidant activity of EGCG in the bladder of BOO rats, we measured MDA level of bladder tissue in the three groups. MDA level is used as an indicator of lipid peroxidation, as lipids in the cell membrane are destroyed to generate MDA in an amount proportional to the degree of tissue destruction. Our results showed that BOO significantly increased the level of MDA in the bladder. It was also showed in our results that the increase in the level of MDA was significantly reduced in BOO rats treated with EGCG which corresponds with other antioxidants [9]. We found that the activities of some redox status markers, such as GSH-Px and total SOD, were significantly decreased in the BOO group compared with the sham group which is similar to the forecast result and other literature [10], and EGCG significantly enhanced the activities of these markers in BOO rats. Our data also showed that EGCG could upregulate CAT activity and downregulate ROS induced by BOO compared with the sham group. It was worth noting that increase of CAT activity in bladder tissue of BOO rats after 4 weeks was similar to our previous study [22], while not all match the other report that CAT activity increased after 2–4 weeks of obstruction, but it decreased markedly in the rabbit bladder with decompensation 8 weeks after partial bladder outlet obstruction (PBOO) [7], which need us to further explore. All the results may indicate that EGCG could alleviate oxidative stress by increasing the activities of antioxidative enzymes in BOO rats.

It is well known that cell apoptosis plays a vital role in leading to bladder dysfunction in BOO model. In our study, TUNEL staining results manifested that a significant reduction of the number of apoptotic cells was found in the treatment of EGCG in BOO rats; it is also found that the expression of caspase-3 in the bladder was decreased in BOO rats when treated with EGCG. These results suggested that EGCG as an antioxidant might protect bladder against BOO-induced apoptosis via decreasing the expression of caspase-3. Meanwhile, the effect of EGCG on cell proliferation by PCNA staining was also measured. Results showed that the cell proliferation level was significantly higher in the bladder of BOO rats with EGCG treatment.

EGCG reported recently can exert a protective effect on rats with obstructive nephropathy by activating the Nrf2 signaling pathway. The H_2_O_2_-exposed hMSCs showed cellular senescence with significantly increased protein levels of acetyl-p53 and acetyl-p21 in comparison with the control hMSCs which was prevented by pretreatment with EGCG [21, 23]. By contrast, in Nrf2-knockdown hMSCs, EGCG lost its antioxidant effect, exhibiting high levels of acetyl-p53 and acetyl-p21 following EGCG pretreatment and H_2_O_2_ exposure. This indicates that Nrf2 may be involved in the antisenescent effect of EGCG in hMSCs [10]. Taken together, these findings suggested the important role of EGCG in preventing oxidative stress-induced cellular senescence and apoptosis through Nrf2 activation. Antioxidants could ameliorate bladder dysfunction in BOO rats according to our previous study [10]; the effect of EGCG on ameliorating bladder dysfunction in BOO model and changing the Nrf2-ARE signaling pathway was investigated in present study. By immunohistochemical staining and Western blotting, we found that the expression of Nrf2 protein in the bladder of BOO rats with EGCG treatment was significantly increased compared to BOO rats. In addition, the level of Nrf2 in the nucleus was markedly increased in the BOO rats when treated with EGCG. The result indicated that EGCG could promote the transportation of Nrf2 into nucleus and the transcription of its target antioxidant genes. We next measured the expression level of HO-1 and NQO1, the downstream genes of Nrf2-ARE pathway by Western blotting. The levels of HO-1 and NQO1 increased significantly in BOO rats after EGCG treatment. Above all, our study suggested that EGCG could promote Nrf2's translocation into the nucleus and the expression of its target antioxidant genes, which ameliorated the oxidative stress in BOO rats.

In conclusion, our study suggested that EGCG has significant protective effects against oxidative stress and could ameliorate bladder dysfunction which may be activation of the Nrf2-ARE pathway and suppressing cellular apoptosis in the bladder of BOO rats.

## Figures and Tables

**Table 1:** Outcomes of cystometric parameters in conscious rats.

	Sham	BOO	BOO + EGCG
Peak voiding pressure	23.54 ± 2.13	42.4 ± 1.44^∗^	55.16 ± 2.6^∗#^
Capacity (ml)	1.15 ± 0.31	3.33 ± 0.38^∗^	4.12 ± 0.49^∗^
Compliance (*μ*l/cm H_2_O)	24.37 ± 3.12	13.17 ± 3.26^∗^	21.36 ± 2.75^#^
Micturition interval (min)	3.67 ± 0.64	2.07 ± 0.47^∗^	3.03 ± 1.41^∗#^

^∗^Significantly different versus the sham group (*P* < 0.05). ^#^Significantly different versus the BOO group (*P* < 0.05).

## Abbreviations

ARE: Antioxidant response element
BOO: Bladder outlet obstruction
BPH: Benign prostatic hyperplasia
GSH-Px: Glutathione peroxidase
CAT: Catalase activity
H&E: Hematoxylin and eosin
HO-1: Heme oxygenase-1
LUTS: Lower urinary tract symptoms
MDA: Malondialdehyde
NQO1: NAD(P)H : quinone oxidoreductase 1
Nrf2: Nuclear erythroid-related factor 2
PE-50: Polyethylene tubing 50
ROS: Reactive oxygen species
EGCG: Epigallocatechin-3-gallate
SOD: Superoxide dismutase
TUNEL: TdT-mediated dUTP nick end labeling.

## References

[1] Ruud Bosch J. L. H., Hop W. C. J., Kirkels W. J., Schröder F. H. (1995). Natural history of benign prostatic hyperplasia: appropriate case definition and estimation of its prevalence in the community. *Urology*.

[2] Fitzpatrick J. M. (2006). The natural history of benign prostatic hyperplasia. *BJU International*.

[3] Madersbacher S., Alivizatos G., Nordling J., Sanz C. R., Emberton M., de la Rosette J. J. M. C. H. (2004). EAU 2004 guidelines on assessment, therapy and follow-up of men with lower urinary tract symptoms suggestive of benign prostatic obstruction (BPH guidelines). *European Urology*.

[4] Oelke M., Kirschner-Hermanns R., Thiruchelvam N., Heesakkers J. (2012). Can we identify men who will have complications from benign prostatic obstruction (BPO)? ICI-RS 2011. *Neurourology and Urodynamics*.

[5] Minciullo P. L., Inferrera A., Navarra M., Calapai G., Magno C., Gangemi S. (2015). Oxidative stress in benign prostatic hyperplasia: a systematic review. *Urologia Internationalis*.

[6] Nocchi L., Daly D. M., Chapple C., Grundy D. (2014). Induction of oxidative stress causes functional alterations in mouse urothelium via a TRPM8-mediated mechanism: implications for aging. *Aging Cell*.

[7] Guven A., Kalorin C., Onal B. (2007). Novel biomarkers of bladder decompensation after partial bladder obstruction. *Neurourology and Urodynamics*.

[8] Zelko I. N., Mariani T. J., Folz R. J. (2002). Superoxide dismutase multigene family: a comparison of the CuZn-SOD (SOD1), Mn-SOD (SOD2), and EC-SOD (SOD3) gene structures, evolution, and expression. *Free Radical Biology & Medicine*.

[9] Zhang B., Wang B., Cao S., Wang Y. (2015). Epigallocatechin-3-gallate (EGCG) attenuates traumatic brain injury by inhibition of edema formation and oxidative stress. *The Korean Journal of Physiology & Pharmacology*.

[10] Hsieh J. T., Kuo K. L., Liu S. H. (2016). Epigallocatechin gallate attenuates partial bladder outlet obstruction-induced bladder injury via suppression of endoplasmic reticulum stress-related apoptosis—in vivo study. *Urology*.

[11] Kanlaya R., Khamchun S., Kapincharanon C., Thongboonkerd V. (2016). Protective effect of epigallocatechin-3-gallate (EGCG) via Nrf 2 pathway against oxalate-induced epithelial mesenchymal transition (EMT) of renal tubular cells. *Scientific Reports*.

[12] Oliva J., Bardag-Gorce F., Tillman B., French S. W. (2011). Protective effect of quercetin, EGCG, catechin and betaine against oxidative stress induced by ethanol in vitro. *Experimental and Molecular Pathology*.

[13] Matsumoto S., Watanabe M., Hashizume K. (2014). Effects of chronic treatment with cilostazol, a phosphodiesterase 3 inhibitor, on female rat bladder in a partial bladder outlet obstruction model. *Urology*.

[14] Gomez-Pinilla P. J., Pozo M. J., Camello P. J. (2007). Aging impairs neurogenic contraction in guinea pig urinary bladder: role of oxidative stress and melatonin. *American Journal of Physiology-Regulatory, Integrative and Comparative Physiology*.

[15] Kregel K. C., Zhang H. J. (2007). An integrated view of oxidative stress in aging: basic mechanisms, functional effects, and pathological considerations. *American Journal of Physiology-Regulatory, Integrative and Comparative Physiology*.

[16] Nomiya M., Andersson K. E., Yamaguchi O. (2015). Chronic bladder ischemia and oxidative stress: new pharmacotherapeutic targets for lower urinary tract symptoms. *International Journal of Urology*.

[17] Camões J., Coelho A., Castro-Diaz D., Cruz F. (2015). Lower urinary tract symptoms and aging: the impact of chronic bladder ischemia on overactive bladder syndrome. *Urologia Internationalis*.

[18] Isbrucker R. A., Edwards J. A., Wolz E., Davidovich A., Bausch J. (2006). Safety studies on epigallocatechin gallate (EGCG) preparations. Part 2: dermal, acute and short-term toxicity studies. *Food and Chemical Toxicology*.

[19] Tay P., Tan C., Abas F., Yim H., Ho C. (2014). Assessment of extraction parameters on antioxidant capacity, polyphenol content, epigallocatechin gallate (EGCG), epicatechin gallate (ECG) and iriflophenone 3-C-*β*-glucoside of agarwood *(Aquilaria crassna)* young leaves. *Molecules*.

[20] Ullmann U., Haller J., Decourt J. D., Girault J., Spitzer V., Weber P. (2004). Plasma-kinetic characteristics of purified and isolated green tea catechin epigallocatechin gallate (EGCG) after 10 days repeated dosing in healthy volunteers. *International Journal for Vitamin and Nutrition Research*.

[21] Zhou P., Yu J. F., Zhao C. G., Sui F. X., Teng X., Wu Y. B. (2013). Therapeutic potential of EGCG on acute renal damage in a rat model of obstructive nephropathy. *Molecular Medicine Reports*.

[22] Liu C., Xu H., Fu S. (2016). Sulforaphane ameliorates bladder dysfunction through activation of the Nrf2-ARE pathway in a rat model of partial bladder outlet obstruction. *Oxidative Medicine and Cellular Longevity*.

[23] Zhang H. S., Wu T. C., Sang W. W., Ruan Z. (2012). EGCG inhibits Tat-induced LTR transactivation: role of Nrf2, AKT, AMPK signaling pathway. *Life Sciences*.

